# Patient-reported outcome measures for hip preservation surgery—a systematic review of the literature

**DOI:** 10.1093/jhps/hnv002

**Published:** 2015-02-06

**Authors:** N. Ramisetty, Y. Kwon, N. Mohtadi

**Affiliations:** 1. Sport Medicine Centre, University of Calgary, 2500 University Drive, Calgary, AB T2N 1N4, Canada; 2. Health Sciences Centre, 3330 Hospital Dr, Calgary, AB T2N 4N1, Canada

## Abstract

Hip preservation surgery is rapidly advancing and patient-reported outcome (PRO) measures are becoming an integral part of measuring treatment effectiveness. Traditionally the modified Harris hip score has been used as the main outcome measure. More recently, new PRO tools in the field have been developed. We performed a systematic review of the English literature from MEDLINE, EMBASE, Cochrane Central Register of Controlled Trials and SPORTDiscus databases to identify the PRO tools used in hip preservation surgery. Our aim was to critically appraise the quality of the questionnaire properties in order to recommend the most appropriate PRO tool for future use. Measurement properties of each PRO questionnaire were rated from excellent to poor, based on Terwee criteria and the results from the included studies. Six PRO tools were identified with description or comparison of their measurement properties in 10 articles. While, most recently developed PRO tools, the hip outcome score (HOS), the Copenhagen hip and groin outcome score (HAGOS) and the international hip outcome tool (iHOT-33) scored better than the others in their measurement properties, iHOT-33 scored the best of all the PRO tools and is recommended for future use in hip preservation surgery.

## INTRODUCTION

There is some evidence for effectiveness of hip arthroscopy both from long-term studies and systematic reviews [1–5]. Most of these existing studies measured outcomes using traditional clinician-administered measures [2–4]. There is little debate that the current standard for measuring the effectiveness of any surgical treatment is to use an outcome that reflects the patient’s perspective [6–8]. These so-called patient-reported outcome (PRO) measures, rather than clinician-administered and surgeon determined objective measures, are critical to the advancement of hip preservation surgery. However, there is no consensus on which PRO to use [9, 10]. Most commonly, the modified Harris hip score (MHHS) has been used in the evaluation of hip arthroscopy outcomes [3, 11]. However, a number of other PRO tools have been developed and head-to-head comparison studies have been published using the new and existing PRO tools [12, 13].

The aim of this study was to perform a systematic review of the English literature of the PRO tools in the hip preservation surgery to identify the available PRO tools in hip preservation surgery and to critically appraise the quality of the questionnaire properties to identify the most appropriate PRO tool that can be used in the future. In order to facilitate the critical appraisal of the review, a brief introduction to the taxonomy describing measurement properties of PRO tools is included.

## MATERIALS AND METHODS

A systematic search was performed to identify the PRO questionnaires used in the hip preservation surgery in young adult population. The following databases were searched electronically from their inception to May 2014: Ovid MEDLINE, Ovid EMBASE, Ovid Cochrane Central Register of Controlled Trials (CENTRAL) and SPORTDiscus. Selected subject headings and keywords were searched on

1. Hip preservation surgery (e.g. hip joint, hip arthroscopy and femoroacetabular impingement).
2. Outcome measurement (e.g. outcome assessment, survey, evaluation, questionnaire).

The resulted articles were subjected to study selection methods as described later to identify relevant articles for the study. Full details of the strategy used to search MEDLINE are provided in the supplementary File S1. It has been modified according to the indexing systems of different databases.

Two reviewers (N.R. and N.M.) independently assessed all retrieved publications from above search, based on the title and abstract. We used inclusion and exclusion criteria as shown in the Table I. If consensus between the two authors was not achieved at this stage, the full article was retrieved. The full articles were assessed again with same inclusion and exclusion criteria to obtain another list of articles. To this list, articles deemed relevant based on previous reviews and the senior author’s expertise, but not identified by the search strategy, were added to result in the final list of included articles for the study. This list included head-to-head comparison studies of PRO questionnaires and studies describing PRO questionnaire measurement properties.

**Table I.** Inclusion and exclusion criteria for study selection

Inclusion criteria	Exclusion criteria
1. Study/article where the main focus was related to the development or evaluation of hip related outcome measures	1. Hip arthroplasty studies
2. The population of interest was patients considered for or who had hip preservation surgery	2. Studies where the population of interest was patients with osteoarthritis
3. Articles published in English language	3. Where the main focus of the study was the clinical outcome rather than the measurement properties of a hip-related PRO measure

Terwee’s *et al.* [14] criteria (described later) for assessing quality of measurement properties were applied to the PRO questionnaires in their respective developmental articles. In addition, the results from the head-to-head comparison studies were analysed. Based on the critical analysis of this collective evidence, measurement properties of each PRO questionnaire were graded from excellent to poor independently by each reviewer (N.R. and N.M.) as per the criteria shown in Table II and recommendations regarding the best PRO tool in hip preservation surgery were made. Differences between the two reviewers were resolved through consensus agreement.

**Table II.** Criteria for summation scoring of PRO questionnaire properties

Excellent	+++	Positive score in all studies
Good	++	Positive score in one study and neutral in others
Fair	+	Positive score in one study and negative in others
Poor	−	Negative score in more than one study or negative score in one study and neutral in others

## AGREEMENT STATISTICS

Agreement statistics were calculated between the two reviewers for study selection criteria using Cohen’s Kappa. The scoring of measurement properties of the outcome measures was evaluated with percent agreement between the reviewers.

## QUALITY ASSESSMENT METHODS FOR OUTCOME MEASURES

There are two separate recognized assessment methods described in the literature for assessing the PRO questionnaires [7, 14].

Mokkink *et al*. [7] developed the Consensus-based Standards for the selection of health Measurement Instruments (COSMIN) checklist for assessing the methodological quality of the articles describing PRO’s. Full details of COSMIN check list are available in their website and article.

Terwee *et al*. [14] developed quality criteria for the measurement properties for PRO questionnaires, the details of which are referred to in their 2007 publication. The quality of each measurement property of the questionnaires are rated as positive (+), intermediate (?), negative (−) or no information available ().

COSMIN checklist was not performed in our study. This was because some of the included PRO questionnaires were developed before COSMIN checklist was introduced and it was felt that, should COSMIN checklist be used, these PRO tools would be at a disadvantage [15–18].

## TAXONOMY OF MEASUREMENT PROPERTIES OF PRO MEASURES

There is no worldwide agreement regarding the terminology to describe the measurement properties of a PRO measure. Mokkink *et al*. [19] undertook a consensus study using the Delphi method with experts in the field: ‘to clarify and standardize terminology and definitions of measurement properties’. The proposed terminology is complex to understand but necessary to critically appraise the PRO’s identified. The main properties are summarized in three domains as reliability, validity and responsiveness [19]. Each domain is further subdivided into measurement properties. Interpretability and floor and ceiling effects are other additional properties.

## THE RELIABILITY DOMAIN

The reliability domain is defined as the degree to which the score is free from measurement error and that scores for patients who have not changed are the same for repeated measurements under several conditions [19]. The reliability domain has three measurement properties namely internal consistency, reliability (test re-test, inter-rater, intra-rater) and measurement error [19].

Internal consistency is the degree of interrelatedness among the items [19]. Internal consistency is typically measured by Cronbach’s alpha coefficient. A value between 0.70 and 0.95 is considered ideal [14]. Lower alpha coefficient is reflective of poor internal consistency and higher value i.e. >0.95 is reflective of redundancy.

Second measurement property of the reliability domain is also considered as reliability according to the COSMIN taxonomy [19]. It is the ability of the tool to yield consistent results when tested over time (test re-test), by different persons on the same occasion (inter-rater) or by the same person on different occasions (intra-rater) [19]. Test re-test reliability is assessed by statistical test intra class correlation (ICC) coefficient or weighted Kappa, which ideally should be more than 0.70 [14]. Test re-test reliability is also known as relative measurement error [14].

The third measurement property of the reliability domain is the absolute measurement error, which is defined as the systematic and random error in a patient’s score that is not attributable to true changes in the concept to be measured [19]. This is calculated as standard error of the mean (SEM). This is reflected in determining the smallest detectable change (SDC), which is the minimum change in the score that is detectable, by the PRO tool with out error. SDC is calculated for individual patients (SDC individual) or for group of patients (SDC group). Minimal important change (MIC) is the minimum change in the score considered clinically important by the patient. For assessing measurement error of a PRO, one should be able to determine SDC and MIC and ideally SDC < MIC both at individual and group level [14]. Some authors consider absolute and relative measurement error as reproducibility [14].

## THE VALIDITY DOMAIN

Validity is defined as the extent the instrument measures the outcome; it is intended to measure [19]. Validity domain has three measurement properties namely content validity, construct validity and criterion validity [19].

Content validity is the most important property of a PRO questionnaire [14]. This property refers to the comprehensiveness of the instrument and measures how well the items in a questionnaire represent all relevant patient concerns. It is critical that patients are involved to determine the content; otherwise the content will only reflect clinicians’ or surgeon’s perspectives [20]. The PRO tool should clearly describe its target population and should derive the questionnaire through the item generation and item reduction methods involving patients and the experts appropriately [14].

Construct validity is the degree to which the outcome score is consistent with another relevant score [19]. There should be a hypothesis stating the presumed correlations (positive or negative) between these scores.

Criterion validity is the validity of the questionnaire as compared with a gold standard [19]. There are no gold standards when it comes to the comparison of outcomes in orthopaedic surgery, and hence, this property was not assessed for any of the included questionnaires.

## THE RESPONSIVENESS DOMAIN

Responsiveness is the ability of a questionnaire to detect change in the patient’s condition over time [14, 19]. As per Terwee *et al.* [14] criteria a positive score is given if the SDC < MIC or the responsiveness ratio of more than 1.96. But responsiveness can also be measured by an anchor based method where the change in the score should correlate to a change in global rating of change (GRC) [21]. GRC, for example, consists of five possible responses for patients to indicate their clinical change as much worse, somewhat worse, no change, somewhat better or much better.

## INTERPRETABILITY

Interpretability is similar to absolute measurement error but is more of a qualitative measurement. Interpretability is defined as the degree to which one can assign qualitative meaning to an instruments’ quantitative scores or change in scores [19].

## FLOOR AND CEILING EFFECTS

Floor effect is a phenomenon of achieving scores at the lower end of the scale leaving little room for a change should the patient deteriorate from that point. Ceiling effect is the opposite effect. Floor or ceiling effects are considered to be significant if >15% of subjects score the lowest or highest possible score, respectively [14].

## RESULTS

**Fig. 1.**
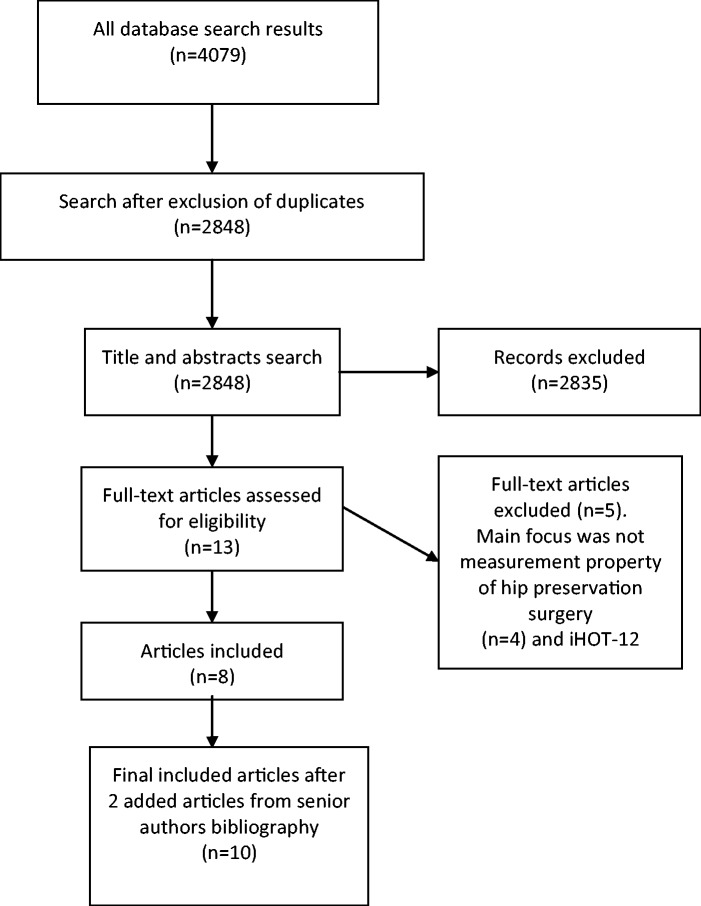
PRISMA flow diagram.

The systematic search resulted in 4079 articles with reduction to 2848 after exclusion of duplicate publications. Title and abstract search based on inclusion and exclusion criteria resulted in 13 articles [12, 13, 15, 16, 21–29]. Full text review excluded four articles where the main focus was not the PRO measurement properties [25–28]. One article evaluated the international hip outcome tool (iHOT)-12 questionnaire and because this PRO represented a subset of the original iHOT-33 questionnaire, it was excluded [29]. To this list two additional relevant articles were added [17, 18]. This search identified six PRO questionnaires (eight articles) and two head-to-head comparison studies [12, 13, 15–18, 21–24]. The preferred reporting items for systematic reviews and meta-analyses (PRISMA) diagram summarizes the search results as shown in Fig. 1.

The final list of 10 articles is shown in Table III. Summary of the common characteristics of the included PRO’s is shown in Table IV. The summation scoring of the PRO questionnaires based on the collective evidence is shown in Table V.

**Table III.** List of included articles for the study (n = 10).

Author	Year published	Questionnaire/type of study	Journal
Christensen *et al*. [15]	2003	NAHS	CORR
Klassbo *et al*. [16]	2003	HOOS	Scand J Rheumatol
*Potter *et al*. [17]	2005	MHHS	Am J Sports Med
*Martin *et al*. [18]	2006	HOS	Arthroscopy
Martin and Philippon [23]	2007	HOS	Arthroscopy
Martin and Philippon [24]	2008	HOS	Arthroscopy
Thorborg *et al*. [21]	2011	HAGOS	Br J Sports Med
Mohtadi *et al*. [22]	2012	iHOT-33	Arthroscopy
Kemp *et al*. [12]	2013	HH	Am J Sports Med
Hinman *et al*. [13]	2014	HH	Br J Sports Med

*Relevant studies not picked up by the search strategy but included in the study. HH—head-to-head comparison study. CORR—clinical orthopaedics and related research

**Table IV.** Common characteristics of included PRO’s

PRO	Number of questions	Subscales	Target population	Score range (worst to best)	Recall period
NAHS	20	4	Young active patients with activity limiting hip pain	0–100	Past 48h
HOOS	40	5	People with hip disability with or with out hip osteoarthritis	0–100	Last week
MHHS	8	2	Patients undergoing hip arthroscopy surgery	0–100	Not available
HOS	28	2	To assess the treatment outcomes of hip arthroscopic surgery	0–100	Last week
HAGOS	37	6	Young to middle-aged physically active patients with hip and/or groin pain	0–100	Last week
iHOT-33	33	4	Young and middle aged active patients with hip disorders	0–100	Last month

**Table V.** Scoring of quality of measurement properties of six PRO’s based on the criteria described in Table II.

PROPERTIES	NAHS	HOOS	MHHS	HOS ADL	HOS sport	HAGOS	iHOT-33
Internal consistency	++	+++	−	++	++	+++	++
Test re-test reliability	+++	+++	+	+++	+++	+++	+++
Content validity	+	+	−	−	−	++	+++
Construct validity	++	++	+++	+++	+++	+++	+++
Responsiveness	N/A	++	++	+++	+	+	+++
Floor or ceiling effects	N/A	−	−	+	+++	−	+++
Interpretability and measurement error	N/A	++	++	+++	+++	++	++

N/A = Information not available.

## AGREEMENT STATISTICS

There was very good agreement between the two reviewers for the study selection methods with a Kappa value of 0.83. Similarly, there was 86% agreement between the two reviewers with respect to the scoring of the measurement properties of the outcome scores.

## DETAILED SUMMARY OF EACH QUESTIONNAIRE

As described in the methodology section earlier, each measurement property of each PRO questionnaire are assessed as per Terwee criteria from their original developmental papers followed by assessment from the results of head-to-head comparison studies of Kemp *et al.* [12] and Hinman *et al.* [13]. A final summation score is then given to each property from excellent to poor as shown in the Table V. As measurement error and interpretability are about assessing similar qualities, they both are assessed together.

## NON-ARTHRITIC HIP SCORE

The non-arthritic hip score (NAHS) was developed for young active patients with higher demands and expectations [15]. This is a patient-based, self-administered questionnaire that was developed as a modification of the Western Ontario and McMaster Universities Osteoarthritis Index (WOMAC) [30]. Designed by Christensen *et al*.[15] the NAHS consists of 20 items distributed in four domains of pain (five items), mechanical symptoms (four items), functional symptoms (five items) and activity level (six items). All 10 questions measuring pain and function come directly from WOMAC index [30]. Input from patients, surgeons, physical therapists and epidemiologists was used in creating NAHS scoring system. Each of the answers corresponds to a particular numerical value (from 0–4) and the values are added at the end of the test and multiplied by 1.25 to arrive at a final score. The maximum score is 100 indicating best hip function.

NAHS is the only PRO questionnaire from our study not included in the head-to-head comparison study by Kemp *et al*. [12]. Hence, the final scoring for the NAHS is based on their original paper from Christensen *et al*. [15] and Hinman *et al*. [13] reliability paper. The NAHS has satisfactory internal consistency with Cronbach’s alpha ranging from 0.69 to 0.92 in each of its four domains [15]. But there is no further evidence about internal consistency from head-to-head comparison studies. Hence, the summation score for internal consistency for NAHS is good. The NAHS has satisfactory reliability with Pearson correlation coefficient ranging from 0.87 to 0.95 for the four subsets and was 0.96 overall [15]. This was further strengthened by the satisfactory ICC of 0.94 noted from the Hinman *et al.* [13] paper. Hence, the summation score for test re-test reliability is excellent.

The NAHS scores fair for content validity. Although patients were involved in the item generation process, the 20 questions included in the PRO tool were somewhat arbitrarily determined without statistical support [6]. This may result in a misrepresentation of items that are relevant to a young, active patient with non-arthritic hip problems [6]. In addition, half of the items were taken directly from the WOMAC index, which were generated in an older, more sedentary population [30].

Construct validity was satisfactory with Pearson correlation coefficients of 0.82 and 0.59 between the NAHS and the Harris hip score (HHS) and Short Form (SF)-12, respectively [15]. But as there was no hypothesis stating the correlations in Christensen *et al.* [15] paper and as there is no further evidence from other studies, the summative score for construct validity is good. There was no information available about responsiveness, floor or ceiling effects and interpretability or measurement error from their original paper.

## HIP DISABILITY AND OSTEOARTHRITIS OUTCOME SCORE

The hip disability and osteoarthritis outcome score (HOOS) was developed as a self-rated evaluative instrument for patients with or without hip osteoarthritis [16]. It is essentially a combination of two previous questionnaires, the WOMAC index and the Knee injury and osteoarthritis outcome score (KOOS) with simply the word hip substituted for the knee [30, 31]. The HOOS 1.1 (39 questions) and the now HOOS 2.0 version has 40 items distributed in five subscales of pain (10 items), other symptoms (five items), function in activities of daily living (ADL; 17 items), function in sport/recreation (four items), hip-related quality of life (QOL;four items). Each question has five scoring options from 0 to 4 in Likert boxes. Scores are summarized for each subscale and transformed to a scale of 0–100 (worst–best).

The HOOS has excellent internal consistency. This was evident from Cronbach’s alpha ranging from 0.77 to 0.96 for all the subscales as described in their original paper [16]. Internal consistency of HOOS was further strengthened and was ranging from 0.91 to 0.96 for all its subscales in Kemp *et al.* [12] paper. HOOS also has excellent test re-test reliability properties. This was evident with ICC ranging from 0.78 to 0.91 for all its subscales from their original paper [16]. Reliability was further strengthened in the Kemp *et al.* [12] paper and was ranging from 0.93 to 0.96 for all its subscales. In addition in the Hinman *et al.* [13] paper, HOOS scored 0.84 to 0.96 for all its subscales.

Patients were involved in the development of the HOOS. But all questions from WOMAC were included irrespective of whether they met the required criteria for inclusion or not in the developmental paper [16]. Hence, some of the items were generated in an older, more sedentary population. These do not reflect patients suitable for hip preservation surgery. Hence, HOOS scores fair for content validity.

There was no information available about construct validity and responsiveness in their original paper [16]. In Kemp *et al.* [12] paper, the correlations with SF-36 subscales was satisfactory and responsiveness for HOOS was satisfactory. Hence, the HOOS scores good for construct validity and responsiveness.

Floor effects were noted in 38% of the items in their original paper [16]. While there were no floor effects for the HOOS in Kemp *et al.* [12] paper, ceiling effects were noted in HOOS ADL (28%) and sport subscales (16%) between 12 and 24 months after surgery. HOOS scores poorly for floor or ceiling effects property as a whole.

There was no information about interpretability, measurement error, MIC and SDC in their original paper [16]. In both the Kemp *et al.* [12] and the Hinman *et al.* [13] papers, the MDC for group and individual level were reported and were at similar ranges for HOOS. In Kemp *et al.* [12] paper, MIC values were reported as well. MIC was noted to be less than MDC at group level, which gives a satisfactory interpretability for HOOS. Hence, overall score for interpretability is good.

## MODIFIED HARRIS HIP SCORE

Originally designed by Harris in 1969, the HHS is a 100-point questionnaire with questions in pain, function, range of motion and deformity [32]. There were 91 points for pain and function and nine points for range of motion and deformity. The modified HHS (MHHS) only includes the pain and function components [33]. The maximum score of 91 is multiplied by 1.1 to give a total score out of 100.

The MHHS score has been widely used in hip arthroscopy surgery [3, 4]. Potter *et al*. [17] compared SF-36 subscales with MHHS. In their study, 33 patients who underwent hip arthroscopy completed SF-36 and MHHS scores. Mean follow-up was 25.7 months. Pearson correlation coefficients for comparing the SF-36 bodily pain, physical function and physical component subscale scores to the MHHS, were 0.73, 0.71 and 0.85, respectively, (*P* < 0.001). They concluded SF-36 demonstrated good correlation with the MHHS for measuring outcomes after arthroscopic labral debridement [17]. This study limits its assessment to only the construct validity for MHHS.

As the MHHS is not prospectively developed for hip preservation surgery, there is lack of information about its measurement properties. This lack of information will be reflected in the final scoring for MHHS on combination with information from Kemp and Hinman papers.

Cronbach’s alpha could not be reported for MHHS from Kemp *et al.* [12] paper. This gives a poor score for MHHS for internal consistency in our summation scoring. ICC for MHHS in Kemp *et al.* [12] paper was satisfactory at 0.91 but not achieved optimum set value of 0.85 in Hinman *et al.* [13] paper with ICC of 0.76. This gives a fair score for test re-test reliability for MHHS. MHHS scores poorly for content validity as this was not aimed at hip preservation surgery population and items were not developed appropriately to score positive as per Terwee *et al.* [14] criteria. Construct validity for MHHS was excellent as noted above and also from Kemp *et al.* [12] paper where satisfactory correlation was noted with SF-36 [17]. Responsiveness was satisfactory for MHHS as per Kemp *et al.* [12] paper and hence scores good. There were no floor effects for MHHS, but ceiling effects were noted in MHHS (24%) between 12 and 24 months after surgery [12]. This gives poor score for MHHS in floor or ceiling effects. Interpretability rating for MHHS is good for same reasons as explained for HOOS.

## HIP OUTCOME SCORE

The hip outcome score (HOS) was developed for patients between the ages of 13 and 66 years [18]. Items were generated by physicians and physical therapists and reduced by factor analysis. The HOS has been described in three papers from 2006 to 2008 [18, 23, 24]. The HOS is a functional measure with no questions related to symptoms [18]. The HOS consists of two functional subscales, ADL and sports: with 19 and nine questions in each subscale, respectively. In addition there are three further questions, which are not utilized towards final score. The questions are rated on a Likert scale from 0 to 4. There is an additional not applicable (N/A) box for patients to tick when their activities were limited by causes other than the hip. So the potential top score is 68 and 36 for ADL and sports subscale, respectively. The scores are divided by highest potential score and multiplied by 100 to achieve a percentage score in each subscale [18].

In their first study, 507 patients with a labral tear were used to determine internal consistency using factor analysis and Cronbach’s alpha coefficients [18]. The second study published in 2007 involved 107 out of 337 patients evaluated retrospectively (mailed questionnaires) who had hip arthroscopy and was completed to expand the validity for the HOS to hip arthroscopy surgery [23]. The third study published in 2008 reported on evidence of reliability and responsiveness for the HOS score [24].

Cronbach’s alpha coefficients were 0.96 and 0.95 for the ADL and sports subscale, respectively, from the HOS paper [18]. Cronbach’s alpha could not be reported for HOS in Kemp *et al.* [12] paper. Hence, final summation score for internal consistency for HOS was considered good.

The ICC for test re-test reliability was satisfactory at 0.98 and 0.92 for ADL and sport subscales, respectively, from its original paper [24]. This was further strengthened in Kemp *et al.* [12] paper where ICC was ranging from 0.95 to 0.96. The optimum ICC for satisfactory test re-test reliability in Hinman *et al.* [13] paper was 0.85. They tested HOS ADL and sports subscale scores and current ADL and sports function. The HOS scored 0.73 to 0.90, falling short of optimum reliability for sport score (0.82) and current ADL function (0.73). Hence, the summation score for ADL and sports subscales for HOS is good.

There was no patient involvement in the development of the HOS [18]. Hence, HOS scores negatively as per Terwee criteria and score poorly at summation scoring. But HOS has an excellent construct validity property. HOS scores positively for construct validity as per their original paper and also scores positively in Kemp *et al.* [12] paper as there was satisfactory correlation noted between HOS and SF-36 [23].

Responsiveness for HOS as described in their paper was satisfactory [24]. In Kemp *et al.* [12] paper, responsiveness for HOS was only satisfactory for ADL subscale but not for sports subscale. Hence, the overall summation score for responsiveness for HOS ADL subscale is excellent and sports subscale is fair.

There were no floor or ceiling effects for HOS in their original papers [24]. While there were no floor effects for the HOS in Kemp *et al.* [12] paper, ceiling effects were noted in the HOS ADL subscale (16%) between 12 and 24 months after surgery. This results in excellent score for sports subscale and fair score for ADL subscale.

The MDC value was three points and MIC values were nine points and six points for ADL and sports subscale scores, respectively, in the HOS paper [24]. In both Kemp *et al.* [12] and Hinman *et al.* [13] paper, MDC for group and individual level were reported and were noted to be slightly higher in the data from Hinman *et al.* [13] paper. In Kemp *et al.* [12] paper, MIC values were reported as well, and MIC was noted to be less than MDC at group level. Hence, overall score for interpretability for HOS is excellent.

## COPENHAGEN HIP AND GROIN OUTCOME SCORE

The Copenhagen hip and groin outcome score (HAGOS) was developed in 2011 and this was the first outcome measure developed with the COSMIN checklist guidelines [21]. HAGOS consists of 37 items distributed in six subscales of pain (10 items), symptoms (seven items), physical function in ADL (five items), physical function in sports and recreation (eight items), participation in physical activities (two items) and hip and/or groin related QOL (five items).

The HAGOS questionnaire was developed in four steps [21]. First step was identifying specific patient population, which was young to middle aged physically active people with hip and/or groin pain. The HAGOS is hence different to other questionnaires in relating the questions for groin problems in addition to hip problems. Second step was the item generation process. They included 43 questions (40 from the HOOS and three from the HOS) based on the evidence from the systematic review of the literature [9]. An expert group of three doctors and four physiotherapists were interviewed going through earlier questions and eight further questions were added. Similar process with 25 patients resulted in addition of two and removal of one question. This resulted in a preliminary 52-item questionnaire. The third and fourth steps were item reduction, which involved 101 patients, and testing of the items for psychometric properties. During this process 14 questions were further removed by the consensus between authors. One further question was removed as a result of factor analysis, resulting in the final 37-item questionnaire [21].

The HAGOS has excellent internal consistency properties. The authors undertook a factor analysis for items, which was described well in their paper [21]. The Cronbach’s alpha ranged satisfactorily from 0.79 to 0.93 for its subscales. This was further strengthened by Kemp *et al.* [12] paper where Cronbach’s alpha was ranging from 0.92 to 0.97.

The HAGOS also has excellent test re-test reliability properties. This was evident from ICC ranging from 0.82 to 0.92 for all its subscales from their original paper [21]. Reliability was further strengthened in the Kemp *et al.* [12] paper and was ranging from 0.92 to 0.97 for all its subscales. In addition in Hinman *et al.* [13] paper, HAGOS scored 0.79 to 0.94 for all its subscales for test re-test reliability.

The HAGOS scores are good for content validity. Patients and experts were involved during item generation and reduction methods. But the major proportion of the questions during item generation was from HOOS with inclusion of all of its 40 questions [21]. Patient group during item generation ended up adding two further questions. Hence, the HAGOS questionnaire reflects closely HOOS questionnaire with few items added and/or deleted in the final questionnaire. Hence, it is possible that the HAGOS may have missed potentially important items inspite of involvement of patients in the item generation phase.

Construct validity was performed as per COSMIN guidelines with priori hypothesis and the results were mostly consistent as per the hypothesis and correlated with SF-36 subscales [21]. This was similar in Kemp *et al.* [12] paper; thereby giving excellent score for construct validity.

The authors measured responsiveness at 4 months from baseline in 87 of the 101 patients [21]. They compared the change scores to asking the patients on a 7-point global perceived effect (GPE) score similar to GRC as described earlier in responsiveness domain. They also measured the standardized response mean (SRM) and effect sizes (ES) on each subscale, which were noticeably higher in patients who had stated that they were ‘much better’ and ‘better’ in their GPE scores. The correlation with GPE score (*r*) is satisfactory with *r* > 0.4 for all subscales [21]. In Kemp *et al.* [12] paper, responsiveness was not satisfactory for HAGOS symptoms, sport and recreation and physical activity subscales (*r* < 0.4). Hence, the summation score for responsiveness for HAGOS is fair.

Floor or ceiling effects were noted in some subscales of HAGOS as described in their original paper [21]. Floor effects were noted for physical activity subscale in 39 and 28% of subjects at baseline and at 4 months, respectively. Ceiling effects were noted for ADL subscale in 18% of subjects at 4 months from baseline. While there were no floor effects for HAGOS in Kemp *et al.* [12] paper, ceiling effects were noted in HAGOS ADL (32%) and physical activity (28%) subscales between 12 and 24 months after surgery. Hence on summation scoring, HAGOS scores poorly for floor or ceiling effects property as a whole.

In the HAGOS original paper, the SDC ranged from 17.7 to 33.8 points at the individual level and from 2.7 to 5.2 points at the group level for the different subscales [21]. The MIC though not clearly defined, was approximated between 10 and 15 points based on the estimate of half of standard deviation (SD). However, since the SDC > MIC at an individual patient level, changes for individual patients may not be easily determined. But this was noted to be similar for other questionnaires as well at individual patient level. In Kemp *et al.* [12] paper, MIC for physical activity level for HAGOS was 1 and it was felt this subscale of HAGOS could not be recommended for research purposes with confidence. Overall summation score for interpretability for HAGOS is good for these reasons.

## INTERNATIONAL HIP OUTCOME TOOL-33

The iHOT-33 was developed with the cooperation of the multi-center arthroscopy of the hip outcomes research network (MAHORN) [22]. The iHOT-33 was designed to address the outcomes of treatment in young active patients with hip disorders (18–60 years old; Tegner activity scale ≥ 4). The study recruited patients from the practices of a group of international hip arthroscopy and arthroplasty surgeons from the United States, Canada, England and Switzerland. The iHOT-33 was created using a process of item generation (51 patients, four orthopaedic surgeons and four physiotherapists), item reduction (150 patients) and pre-testing (31 patients). The questionnaire was tested for test re-test reliability (123 patients), face, content and construct validity (51 patients) and responsiveness over a 6-month period in post-arthroscopy patients (27 patients); for a total of 433 patients [22]. Initially, 146 items identified through patient query to the point of redundancy, were reduced to 60 through item reduction, and categorized into four domains: (i) symptoms and functional limitations, (ii) sport and recreational physical activities, (iii) job-related concerns and (iv) social, emotional and lifestyle concerns and formatted using a visual analogue scale. Pre-testing confirmed appropriate wording, content and formatting. Test re-test reliability showed Pearson correlations greater than 0.80 for 33 of the 60 questions. These 33 questions were formulated into a self-administered questionnaire using a visual analogue scale response format from 0 to 100 (worst–best outcome) [22].

Internal consistency was calculated with Cronbach’s alpha at 0.99 [22]. This very high value suggests likely redundancy of one or more items [14]. But it can also be high if two or more subscales with high alphas are combined. This is the likely cause for high Cronbach’s alpha for iHOT-33 as the determined 0.99 alpha is for the whole score rather than the subscales [22]. Cronbach’s alpha was 0.96 for iHOT-33 in Kemp *et al.* [12] paper. Hence, the summation score for internal consistency for iHOT-33 is good.

The IHOT-33 has excellent test re-test reliability properties. This was evident from ICC value of 0.78 from their original paper [22]. Reliability was further strengthened in Kemp *et al.* [12] paper with an ICC value of 0.93. In addition in Hinman *et al*. [13] paper, iHOT-33 scored 0.86 to 0.93 for all its subscales.

The iHOT-33 has excellent content validity properties. This is based on a number of factors. There were more than 400 patients involved in the development of the questionnaire. Independent groups of patients were used at each stage of the questionnaire development [22]. A large number of items (146) were generated during item generation phase making sure comprehensiveness was ensured by repeated surveying of patients and sampling to the point of redundancy, until no new items were generated. Hence, iHOT-33 represents a true patient generated questionnaire.

Construct validity was demonstrated with a correlation of 0.81 to the NAHS [22]. This was further strengthened by satisfactory correlation with SF-36 in Kemp *et al.* [12] paper. This gives excellent score for construct validity for iHOT-33.

Responsiveness was demonstrated with a responsiveness ratio of 6.7 [22]. This scores positive as per Terwee *et al.* [14] criteria. Responsiveness was satisfactory in Kemp *et al.* [12] paper with high correlation noted (*r* > 0.4) with GRC score. This gives excellent score for responsiveness for iHOT-33.

There were no floor or ceiling effects noted for iHOT-33 in their original paper [22]. In the Kemp *et al.* [12] paper, there were no floor or ceiling effects for iHOT-33. Hence, iHOT-33 scores excellent for floor or ceiling effects.

The MIC for the iHOT-33 was six [22]. Such a low MIC makes the iHOT attractive as an outcome tool in calculating sample sizes for prospective research studies. Although mean and SD values for whole score were known, subscale details were not given in their original paper [22]. Interpretability was strengthened by satisfactory MIC and MDC group values for the iHOT-33 in Kemp *et al.* [12] paper. Hence, the summation score for interpretability for the iHOT-33 is good.

## COMPARISON STUDIES

Kemp *et al*. [12] study published in 2013 looked at and compared the psychometric properties of the commonly used PRO’s including the newer tools except NAHS. They compared five PRO’s including HOOS, MHHS, HOS, HAGOS and iHOT-33 in 50 patients who underwent hip arthroscopy surgery compared with 50 age matched control patients. The hip arthroscopy group completed all the questionnaires on three occasions and control group completed the questionnaire on one occasion. They assessed reliability, validity, responsiveness, interpretability and floor and ceiling effects for all these PRO’s. They conclude that the iHOT-33 and the HOOS are the most appropriate current PRO’s available for hip arthroscopy population.

Hinman *et al*. [13] conducted a recent study in 2014 looking only at test re-test reliability of same six PRO’s identified in this review. They included 30 patients with femoroacetabular impingement (FAI) who filled six questionnaires on two occasions 1–2 weeks apart. They calculated ICC, SEM and MDC. An ICC of 0.85 was set as the optimum target level for reliability. They concluded that the majority of the questionnaires was reliable and precise enough for use at the group level. The exceptions were MHHS and majority of HOS where the reliability point estimates and confident intervals fell below the benchmarks. The measurement error at the individual patient level was larger for all questionnaires compared with the error at the group level.

## DISCUSSION

Traditionally MHHS has been used as the standard PRO questionnaire for hip preservation surgery [4, 11]. Systematic reviews were published in the quest to identify the best PRO tool in the hip preservation surgery [9, 10, 34]. Since the last systematic review by Tijssen *et al*. two other PRO tools were developed [21, 22]. Most recently, there were two published head-to-head comparison studies comparing the relevant PRO tools [12, 13]. To our knowledge, this study is the only systematic review to date including the most recently developed PRO questionnaires [21, 22].

Thorborg *et al*. [9] performed a systematic review in 2010 to determine whether there was a valid, reliable and responsive PRO to assess hip and groin disability. They studied 41 papers covering 13 PRO’s. They included PRO’s for arthritic and non-arthritic hip pathology requiring non-operative treatment, hip arthroscopy or total hip replacement (THR) as well as patients following groin-hernia repair and unspecified hip pain. They recommended HOOS for evaluating patients with hip OA undergoing non-surgical or surgical treatment such as THR and HOS for evaluating patients undergoing hip arthroscopy [9].

Lodhia *et al*. [34] performed a systematic review in 2011 of the psychometric properties for PRO’s for FAI and hip labral pathology. They evaluated HOS, WOMAC and NAHS from five relevant studies. Their assessment of these three PRO’s has shown HOS with high ratings for most clinimetric properties and concluded HOS as the most proven instrument in FAI and labral tears. They failed to emphasize the main drawback of the HOS, which had a negative score for content validity because there was no patient involvement. They qualified their conclusions by recommending that further longitudinal studies were warranted.

Published later in the same year (2011), Tijssen *et al*. [10] performed a review of the psychometric evidence for PRO’s for hip arthroscopy. Their search strategy resulted in five studies covering three PRO’s, the NAHS, the HOS and the MHHS. Their study is unique in that they assessed both the methodological quality of all five studies using COSMIN checklist and also rated each questionnaire psychometric properties based on Terwee criteria. This review was somewhat contradictory to the Lodhia review in that the authors suggested the NAHS was the best quality questionnaire, but the methodological quality of the HOS, as per COSMIN checklist, scored better.

All three earlier systematic reviews were performed before HAGOS and iHOT-33 were developed. Most recently in 2014, Harris-Hayes *et al*. [35] performed a review of the PRO’s in FAI including the newer tools. Their study was not a systematic review. They excluded PRO’s, which did not include patients in the development of the questionnaire thereby excluding HOS and MHHS ensuring adequate content validity. They compared NAHS, HAGOS and iHOT-33. Using COSMIN rating of questionnaire quality, they rated HAGOS and iHOT-33 as the best, but suggested that, more head-to-head comparison studies are required to definitively recommend either or both. The drawback noted for iHOT-33 was that the subscales were not validated for use like the HAGOS and NAHS subscales.

These reviews reflect the lack of agreement that is apparent when making a decision on which questionnaire to use for patients with hip preservation surgery.

Although our study provides a comprehensive overview of PRO tools, there are some limitations. There are only two head-to-head comparison studies using the same population of patients. Hinman *et al*. study assessed the reliability of the six outcomes, whereas Kemp *et al*. study, although evaluating all properties, used only five of the PRO questionnaires. The literature in this review is confined to the English language. The authors are not aware of similar foreign language outcomes but this is certainly possible. There may be a bias towards the iHOT-33 PRO tool in this study, as the senior author of this study is the primary author/developer of the iHOT-33 questionnaire. This bias is negated by the fact that the first author worked independently, assessed all the information prior to final agreement and where disagreement occurred the final decision was weighted to the first author.

## WHICH IS THE BEST PRO TOOL AVAILABLE?

It is clear that rigorous scientific comparison of well-developed questionnaires is a difficult task. As shown, all questionnaires scored well on most properties (Table V). Summating all of the ‘+’ and ‘−’ from this table would be an arbitrary way to rank the questionnaires. A better way would be to understand what are the most important characteristics or at what threshold values would a questionnaire be acceptable. Assuming that the questionnaires have acceptable, internal consistency, reliability, interpretability, no floor or ceiling effects and are responsive to change then the most important characteristic has to be the content validity. Outcomes research, effectiveness trials and indeed prospective studies to determine the best treatments must measure what is important to patients. Therefore, all outcome measures must reflect important and relevant patient characteristics i.e. content. On summation of all the information from the developmental papers of the respective questionnaires using the Terwee criteria, evidence from the Kemp and Hinman comparison papers, the iHOT-33 currently is the best available PRO tool for hip preservation surgery.

## CONCLUSION

This systematic review of the English literature identified six patient-reported outcome tools suggested for use in hip preservation surgery. Critical appraisal of the development, measurement properties and head-to-head comparison studies suggest that the iHOT-33 is the recommended PRO tool for future use in hip preservation surgery.

More well designed, prospectively performed head-to-head comparison studies involving different patient populations, including non-surgical, hip arthroscopic and open hip preservation treatments would provide greater clarification in this field of outcome assessment.

## SUPPLEMENTARY DATA

Supplementary data are available at *Journal of Hip Preservation Surgery* online.

## CONFLICT OF INTEREST STATEMENT

None declared.

## Supplementary Material

Supplementary Data

Supplementary Data
