# Supplementary material for: Patient-reported outcome measures for hip preservation surgery—a systematic review of the literature
Source: J Hip Preserv Surg. 2015 Feb 6;2(1):15–27. doi: 10.1093/jhps/hnv002 (PMC4718480; doi:10.1093/jhps/hnv002)
Supplement: Supplementary Data [file supp_2_1_15__index.html]

Patient-reported outcome measures for hip preservation surgery—a systematic review of the literature — Patient-reported outcome measures for hip preservation surgery—a systematic review of the literature — Supplementary Data 

# Patient-reported outcome measures for hip preservation surgery—a systematic review of the literature

## Supplementary Data

files

**Files in this Data Supplement:**

- Supplementary Data - docx file
