# Supplementary material for: Patient-reported outcome measures for hip preservation surgery—a systematic review of the literature
Source: J Hip Preserv Surg. 2015 Feb 6;2(1):15–27. doi: 10.1093/jhps/hnv002 (PMC4718480; doi:10.1093/jhps/hnv002)
Supplement: Supplementary Data [file supp_hnv002_supplementary_file_1.docx]

MEDLINE search strategy

1. "Hip Joint"/pa [Pathology]

2. hip pathology.tw.

3. hip pain.tw.

4. (hip and groin pain).tw.

5. exp Hip Joint/ and exp Arthroscopy/

6. (hip adj2 arthroscop*).tw.

7. hip preserv*.tw.

8. femoroacetabular impingement.tw.

9. femoro acetabular impingement.tw.

10. exp Femoracetabular Impingement/

11. or/1-10

12. exp Treatment Outcome/

13. exp "Outcome Assessment (Health Care)"/

14. outcome?.tw.

15. survey?.tw.

16. evaluation?.tw.

17. exp Self-Assessment/

18. self assessment.tw.

19. exp Questionnaires/

20. questionnaire?.tw.

21. "Self Report"/

22. self report*.tw.

23. patient report*.tw.

24. (score or scoring).tw.

25. "Pain Measurement"/

26. validity.tw.

27. or/12-26

28. 11 and 27

29. exp Aged/

30. (elder* or geriatric*).af.

31. (old* adj (person or adult* or people or patient? or inpatient? or outpatient?)).af.

32. or/29-31

33. (athelete? or player?).af.

34. (adolescen* or teen* or youth? or minor?).af.

35. (young* adj (person or adult* or people or patient? or inpatient? or outpatient?)).af.

36. middle aged.af.

37. or/33-36

38. 28 and 32

39. 28 and 32 and 37

40. 38 not 39

41. 28 not 40
